# Supplementary material for: Implementation and evaluation of an antimicrobial stewardship programme in companion animal clinics: A stepped-wedge design intervention study
Source: PLoS One. 2019 Nov 18;14(11):e0225124. doi: 10.1371/journal.pone.0225124 (PMC6860428; doi:10.1371/journal.pone.0225124)
Supplement: Attachment — Submitted filename: PLoS One ASP.docx [file pone.0225124.s001.docx]

This study assesses the impact of an antimicrobial stewardship program (ASP) on prescribing practices in 41 Dutch companion animal practices. ASP has been relatively neglected in companion animals globally compared to the focus on food animals, but is an important topic because people interact more closely with pet than with food animals (or their products). As expected from this group, the study was very well designed, in this case using a stepped-wedge intervention study implementing an ASP which was specifically developed for this study, and used practice management software to obtain the monthly data on antimicrobial use (AMU) needed (cats, dogs, rabbits) 2-years prior and during the 12-month ASP study period. The ASP program used a “RESET” model to guide changes in prescribing behaviour. The study showed a significant reduction in AMU and a significant shift to use of AMs of lower importance (except for third choice which were already low). There were considerable clinic differences. The discussion is excellent, self-critical of the limitations but with excellent arguments for the validity of the findings. The discussion is an excellent guide to others in other countries contemplating this type of needed action. This article leads the way in an on objective science-based intervention and analysis. The writing, tables, and figures are uniformly excellent.

Line 292: net spelling

302: The goal of ….
